# Cerebro- and Cardio-vascular Responses to Energy Drink in Young Adults: Is there a Gender Effect?

**DOI:** 10.3389/fphys.2016.00346

**Published:** 2016-08-10

**Authors:** Cathríona R. Monnard, Jean-Pierre Montani, Erik K. Grasser

**Affiliations:** Department of Medicine/Physiology, University of FribourgFribourg, Switzerland

**Keywords:** cerebral blood flow velocity, beat-to-beat measurements, caffeinated beverage, women, red bull

## Abstract

**Background and Purpose:** Energy drinks (EDs) are suspected to induce potential adverse cardiovascular effects and have recently been shown to reduce cerebral blood flow velocity (CBFV) in young, healthy subjects. Gender differences in CBFV in response to EDs have not previously been investigated, despite the fact that women are more prone to cardiovascular disturbances such as neurocardiogenic syncope than men. Therefore, the aim of this study was to explore gender differences in cerebrovascular and cardiovascular responses to EDs.

**Methods:** We included 45 subjects in a retrospective analysis of pooled data from two previous randomized trials carried out in our laboratory with similar protocols. Beat-to-beat blood pressure, impedance cardiography, transcranial Doppler, and end-tidal carbon dioxide (etCO_2_) measurements were made for at least 20 min baseline and for 80 min following the ingestion of 355 mL of a sugar-sweetened ED. Gender and time differences in cerebrovascular and cardiovascular parameters were investigated.

**Results:** CBFV was significantly reduced in response to ED, with the greatest reduction observed in women compared with men (−12.3 ± 0.8 vs. −9.7 ± 0.8%, *P* < 0.05). Analysis of variance indicated significant time (*P* < 0.01) and gender × time (*P* < 0.01) effects. The percentage change in CBFV in response to ED was independent of body weight and etCO_2_. No significant gender difference in major cardiovascular parameters in response to ED was observed.

**Conclusions:** ED ingestion reduced CBFV over time, with a greater reduction observed in women compared with men. Our results have potential implications for women ED consumers, as well as high-risk individuals.

## Introduction

Energy drink (ED) consumption has increased over the past decade and with it an increasing number of studies have reported adverse cardiovascular events associated with these drinks (Grasser et al., 2014; Svatikova et al., 2015), such that researchers caution against the use of EDs among children, pregnant women and those with a history of cardiovascular conditions (Higgins et al., 2015). A recent systematic review highlighted potential detrimental effects of ED consumption, particularly in predisposed individuals and in those undergoing treatment with drugs known to modulate cardiac impulse propagation, by affecting cardiovascular and neurological systems (Ali et al., 2015). Moreover, EDs have also been shown to acutely increase blood pressure, heart rate and cardiac output, as well as alter electrolyte balance and induce repolarization abnormalities (Kozik et al., 2016) and EDs have recently been linked to a positive inotropic effect on the heart, including increasing heart rate, cardiac output and contractility, stroke volume and blood pressure (Lippi et al., 2016). In addition, EDs may favor endothelial dysfunction and platelet aggregation, as well as increase glycemia, total cholesterol and LDL-cholesterol (Lippi et al., 2016). Together these biological abnormalities have been suggested to mediate cardiovascular dysfunction (Lippi et al., 2016).

Despite the fact that EDs are often promoted for their ability to increase mental performance, recent studies have shown alterations in cerebrovascular function, particularly cerebral blood flow velocity (CBFV), whereby ingestion of an ED acutely reduced CBFV in healthy, young men and women (Grasser et al., 2014, 2015). It is well known that gender differences in CBFV exist under resting conditions, which are suggested to originate from physiological and anatomical differences between men and women (Ackerstaff et al., 1990; Marinoni et al., 1998; Nagai et al., 1998). However, it is not known whether CBFV differs between genders in response to EDs. Moreover, gender differences in hemodynamic response to EDs have not previously been reported, despite the fact that the incidence of neurocardiogenic syncope in women is twice that of men (Colman et al., 2004).

To the best of our knowledge, two recent studies from our laboratory are the only studies that have previously documented changes in CBFV in response to ED (Grasser et al., 2014, 2015). However, the sample size of both studies was too small to investigate differences between genders. Therefore, the rationale for this study was to combine both studies and conduct an analysis in order to investigate possible gender differences in a larger cohort by exploring their cerebro- and cardio-vascular responses to ED.

## Materials and methods

We carried out a retrospective analysis of data from two previously reported studies from our laboratory (Grasser et al., 2014, 2015). Pooled data from the two studies gave a total of 45 (22 women) healthy, young subjects.

Subjects were students recruited from the university population and their friends. Individuals with any pre-existing disease or who were taking any medication affecting cardiovascular regulation, as well as competition athletes and individuals with a daily exercise workload exceeding 60 min per day, were excluded from the studies. All participants fasted for ≥12 h and abstained from alcohol, smoking and caffeine, as well as from vigorous exercise for 24 h before each test and were advised not to change their diet between tests. Both studies were conducted according to the guidelines laid down in the Declaration of Helsinki and the joint ethical committee of the States of Jura, Fribourg and Neuchâtel approved all procedures involving human subjects. All subjects provided written consent prior to the start of their studies.

All experiments were performed in a quiet, temperature-controlled (~22°C) laboratory and commenced between 08:00 and 09:00 AM. Subjects attended two separate experimental visits (each separated by at least 2 days) according to a randomized cross-over design (ED versus water negative control). On arrival in the laboratory, subjects were asked to empty their bladders if necessary and proceeded to sit in a comfortable chair with armrests. Equipment for cardiovascular and cerebrovascular recordings was then attached. After a period of stability (approximately 30 min), baseline recordings were taken for at least 20 min. Following this, subjects ingested, in a non-blinded fashion, either 355 ml of degassed Red Bull ED containing caffeine (~114 mg), sucrose and glucose (~39.1 g), taurine (~1420 mg), and glucuronolactone (~84.2 mg) or 355 ml tap water at room temperature. A 4 min period was provided to drink at a convenient pace. Post-drink recordings were carried out and data for the first 80 min were pooled since both of our previous studies had similar protocols during the first 80 min with the peak hemodynamic response reached around 60–80 min. In addition, gender comparison between the two original studies revealed no significant differences for age, height, weight or BMI (all *P* > 0.05), which justified merging the two data sets.

CBFV_mean_ was measured continuously using transcranial Doppler ultrasonography (Doppler-Box, DWL, Sipplingen, Germany), whilst end-tidal carbon dioxide (etCO_2_) was measured with a nasal cannula by infrared absorption and breathing frequency (BF) was calculated automatically from the CO_2_ curve for each breathing cycle. Cardiovascular and electrocardiographic (cardiac intervals) recordings were performed beat-to-beat using a Task Force Monitor (CNSystems, Medizintechnik, Graz, Austria) and were merged real-time with CBFV recordings; data were sampled at 1000 Hz.

Cerebrovascular resistance index (CVRI) was calculated as the mean blood pressure (MAP) at brain level divided by CBFV_mean_. Heart rate (HR) was calculated from the appropriate RR-Interval. Cardiac output (CO) was computed as the product of stroke volume (SV) and HR. MAP was calculated from diastolic blood pressure (DBP) and systolic blood pressure (SBP) as follows: MAP = DBP + 1/3 (SBP − DBP). Total peripheral resistance (TPR) was calculated as MAP/CO.

Data were analyzed using Statistix software (Version 8, Tallahassee, Florida, USA). Independent *t*-tests were performed to explore gender differences. Two-way repeated measures ANOVA was performed with time as within subject factor and gender as between subject factor. Linear regression analysis was carried out to determine significant predictors of gender effects. ANCOVA was used to determine gender differences in CBFV while controlling for respective baseline differences. All reported *P*-values are two-sided and statistical significance was set at a level of *P* < 0.05. Data are presented as mean ± standard error (SE).

## Results

Subject characteristics from the current analysis are presented in Table 1. When comparing men and women, men were significantly older, taller and heavier than women (Table 1). We observed significant baseline gender differences in CBFV, CVRI, BF, SBP, and TPR, but not for etCO_2_, DBP, HR, SV, or CO (Table 2).

**Table 1:** **Subject characteristics of 23 men and 22 women**.

	**Men**	**Women**	**All**
Age, years	23.2 ± 0.7^*^	21.4 ± 0.3	22.3 ± 0.5
Height, cm	179 ± 1^***^	167 ± 1	173 ± 1
Weight, kg	75 ± 2^***^	61 ± 1	68 ± 1.5
Body mass index, kg •m^−2^	23.4 ± 0.6	21.8 ± 0.5	22.5 ± 0.6

* P < 0.05 and

*** P < 0.005, significant difference comparing men and women using an unpaired t-test.

**Table 2:** **Cerebro- and cardio-vascular responses to energy drink (ED) consumption**.

	**Men**		**Women**		ANOVA Interaction effect (gender^*^time)	***ANOVA Time effect***
	**Baseline ED**	**Δ Responses ED^#^**	**Baseline ED**	**Δ Responses ED^#^**		
Cerebral blood flow velocity, cm • s^−1^	56 ± 3^***^	−5.6 ± 0.6^***^	72 ± 4	−9.0 ± 0.9	< 0.01	< 0.005
Cerebral vascular resistance index, mmHg • s • cm^−1^	1.6 ± 0.1^***^	+0.2 ± 0.0	1.2 ± 0.1	+ 0.2 ± 0.0	0.69	< 0.005
End-tidal carbon dioxide, mmHg	38.5 ± 0.6	−0.6 ± 0.2^*^	36.9 ± 0.6	−1.4 ± 0.2	< 0.01	< 0.005
Breathing frequency, breaths • min^−1^	14.4 ± 0.6^*^	+1.1 ± 0.3	16.4 ± 0.5	+1.3 ± 0.2	0.60	< 0.005
Systolic BP, mmHg	119 ± 2^***^	+2.3 ± 0.9	112 ± 2	+3.7 ± 1.1	0.80	< 0.005
Diastolic BP, mmHg	76 ± 1	+2.4 ± 0.7	73 ± 2	+2.4 ± 0.8	0.51	< 0.005
Heart rate, beats • min^−1^	61 ± 2	+1.9 ± 0.6	62 ± 2	+1.1 ± 0.7	0.34	< 0.005
Stroke volume, mL	82 ± 3	+2.6 ± 0.8	84 ± 3	+2.6 ± 0.9	0.14	< 0.005
Cardiac output, L • min^−1^	5.0 ± 0.1	+0.3 ± 0.1	5.2 ± 0.2	+ 0.4 ± 0.1	0.23	< 0.005
Total peripheral resistance, mmHg • min • L^−1^	18.5 ± 0.4^*^	−0.6 ± 0.3	17.0 ± 0.6	−0.2 ± 0.3	0.53	0.04

BP: Blood pressure;

# mean responses over 80 min compared to baseline values equivalent to the area under curve presented as a delta;

* P < 0.05 and

*** P < 0.005, significant different difference comparing men and women using an unpaired t-test.

**Figure 1:**
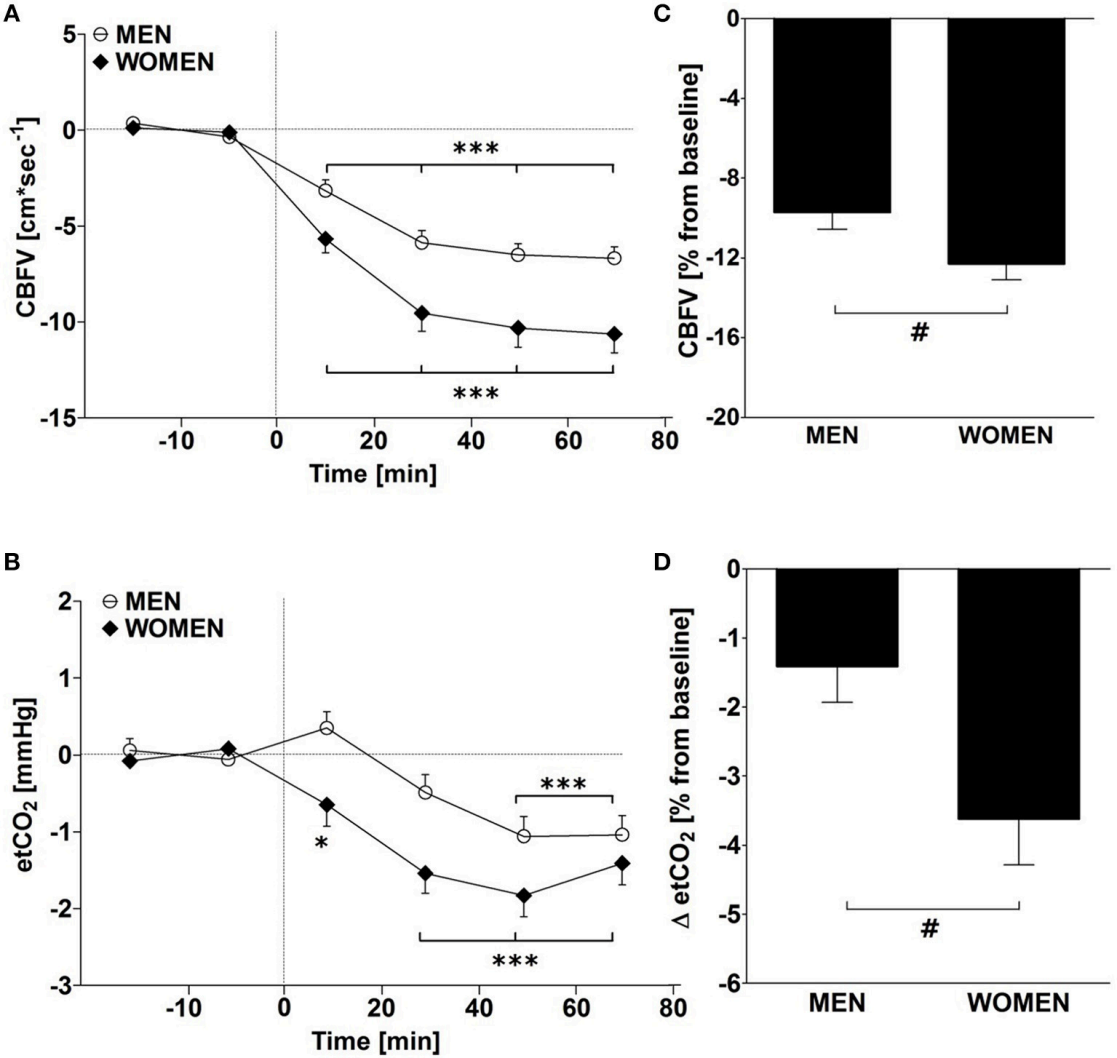
**(A,B)** Time course changes for cerebral blood flow velocity (CBFV) and end-tidal carbon dioxide (etCO_2_) after ingestion of 355 ml of an energy drink in 23 men (open circle) and 22 women (closed rhombus). ^*^P < 0.05, ^***^P < 0.005, statistically significant differences over time from baseline values using repeated measures ANOVA with Dunnett's multiple comparison *post-hoc* test. **(C,D)** Mean changes over 80 min post-drink and presented as a percentage change relative to baseline values in men and women. **#***P* < 0.05, statistically significant difference between CBFV responses in men and women using an unpaired *t*-test.

**Figure 2:**
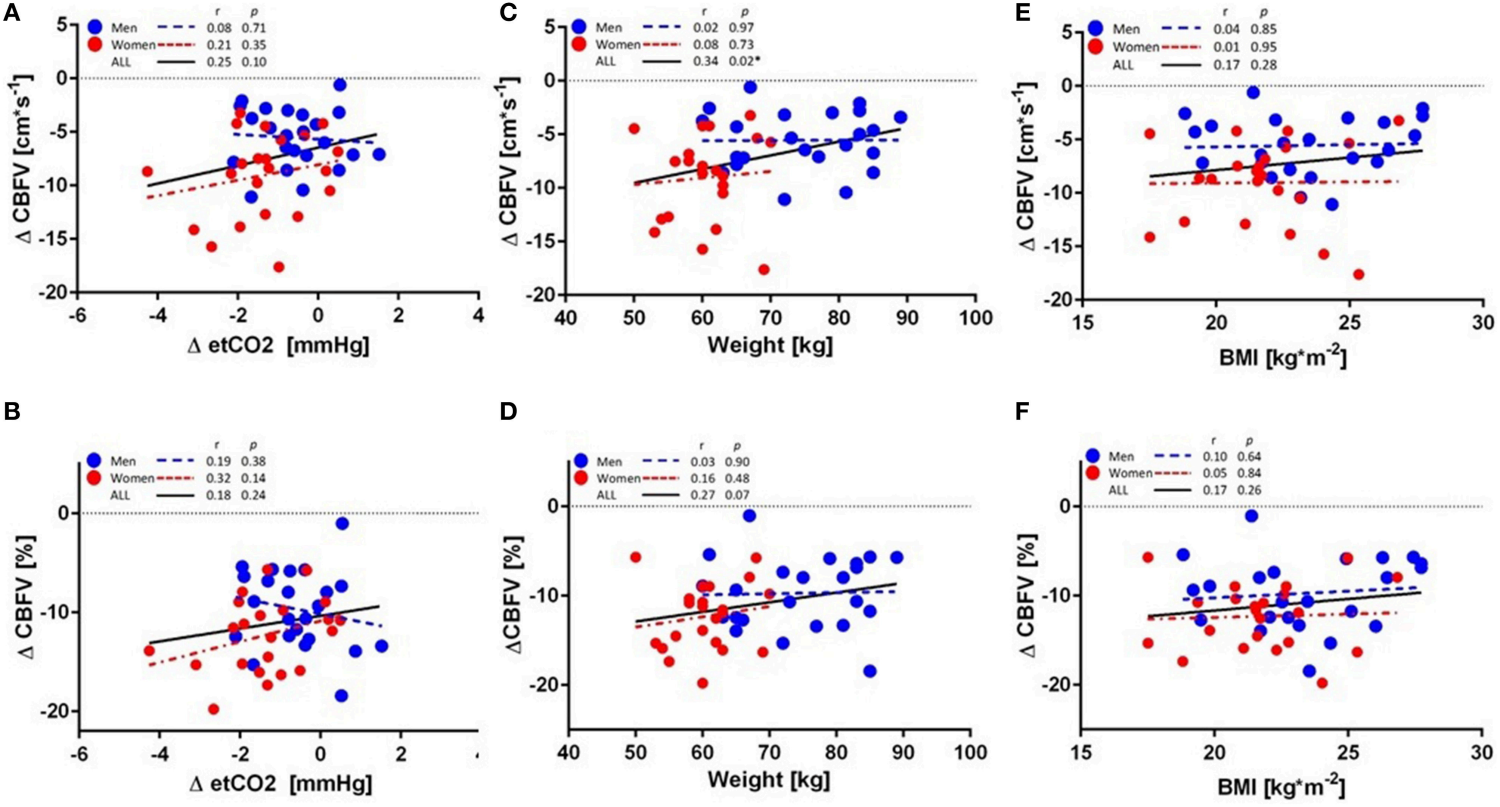
**Linear regression analyses of (A) absolute changes in cerebral blood flow velocity (CBFV) and end-tidal carbon dioxide (etCO_2_), (B) percentage changes in CBFV and etCO_2_, (C) absolute change in CBFV and body weight, (D) percentage changes in CBFV and body weight, (E) absolute changes in CBFV and BMI, (F) percentage changes in CBFV and BMI in 23 men (blue circle) and 22 women (red circle)**. Regression lines for all subjects (black), men (blue), and women (red) are highlighted. ^*^P < 0.05, statistically significant correlation.

In response to ED consumption, no significant gender differences in the major hemodynamic variables (SBP, DBP, HR, SV, CO, TPR) were found (*P* > 0.05, all; Table 2). CVRI and BF were also not significantly different between genders (*P* > 0.05, all). In contrast, there were significant gender differences in the reductions in CBFV and etCO_2_ with a greater response in women (Figures 1A,B; Table 2). Linear regression analysis revealed a significant influence of baseline CBFV on changes in CBFV (*r* = 0.7, *P* < 0.01). However, even when expressed as a percentage of the baseline value, women showed a greater reduction in CBFV than men in response to ED (−12.3 vs. −9.7 %, *P* < 0.05; Figure 1C). Similarly, women also showed a significantly greater reduction in etCO_2_ as a percentage of baseline compared with men (−3.6 vs. −1.4 %, *P* < 0.05; Figure 1D). However, no significant correlation was observed between the absolute and % changes in CBFV and changes in etCO_2_ among all subjects, as well as for men and women, separately (Figures 2A,B).

The potential effect of anthropometric variables on CBFV was also examined. A significant correlation was observed between changes in CBFV and body weight among all subjects (*r* = 0.34, *P* = 0.02; Figure 2C), but not for men (*r* = 0.02, *P* = 0.97) and women (*r* = 0.08, *P* = 0.73) separately, and the regression slopes were not significantly different between men and women (*P* = 0.73). No correlation was found between % change in CBFV and body weight for any group (Figure 2D). No significant correlation was observed between absolute change in CBFV or % change in CBFV and BMI for any group (Figures 2E,F).

Stepwise linear regression was carried out using the change in CBFV as dependent variable and baseline CBFV, CVRI, BF, SBP, and TPR, as well as the change in etCO_2_ and weight as independent variables. Results showed that baseline CBFV was the only significant predictor of the change in CBFV, while all other variables were dropped from the regression model. Further analysis controlling for baseline CBFV using ANCOVA diminished the gender response, but it nonetheless remained significantly greater in women than in men (*P* < 0.05).

## Discussion

ED consumption resulted in a significant reduction in CBFV in young, healthy subjects with a greater CBFV reduction in women compared with men. This gender difference remained significant even after adjusting for baseline differences in CBFV.

Our finding of gender differences in baseline CBFV is in line with previous investigations showing higher middle cerebral artery (MCA) flow velocity in women compared with men (Ackerstaff et al., 1990; Marinoni et al., 1998), which was age-independent (Ackerstaff et al., 1990). Similarly, Nagai et al. demonstrated significantly higher systolic and diastolic flow velocities in women compared with men in the MCA and the internal carotid artery (Nagai et al., 1998). Several factors may contribute to these observations. The lower hematocrit in women compared with men may lead to greater blood flow per 100 g of brain tissue in order to compensate for the decreased oxygen transport capacity to the metabolically active organ. In addition, the anatomically smaller arterial size in women compared to men translates, for the same amount of blood flow, into a greater flow velocity (Nagai et al., 1998).

The reduction in CBFV in response to ED might result from the caffeine contained in the ED, as previous studies have demonstrated reductions in cerebral blood flow (CBF) in response to caffeine ingestion (Mathew and Wilson, 1985; Addicott et al., 2009). In our study, men and women received the same dose of ED, but since women are smaller in body weight one might expect a higher concentration of circulating caffeine in the bloodstream and thus a greater response to ED. However, when data for men and women were analyzed separately, no significant correlation was observed for CBFV and body weight, which suggests that there was no dose effect. In fact, the observation that the regression lines for men and women were nearly horizontal and not significantly different, but with a line shifted downward and to the left for women (Figures 2C,D), suggests that factors other than body weight may contribute to the greater CBFV response in women. Given that etCO_2_ has been shown to be a determinant of cerebral blood flow (CBF) (Mathew et al., 1986), and that we identified a significant gender difference in etCO_2_ in response to ED, we then explored whether this change in etCO_2_ may play a role in the observed changes in CBFV. However, although ED ingestion led to a greater decrease in etCO_2_ in women on average, we found no significant association between changes in CBFV and changes in etCO_2_, which is in line with a previous report showing that decreases in CBF in response to caffeine were not associated with changes in etCO_2_ (Mathew and Wilson, 1985).

In addition to caffeine, the ED used in this study contained ~39.1 g of sugar, which may have contributed to the change in CBFV. In this context, sugar has been shown to modulate CBF, as demonstrated by Page et al. (2013), who showed a greater reduction in hypothalamic CBF following ingestion of 75 g of glucose vs. fructose (−5.45 vs. 2.84 mL/g per minute, respectively). A more recent study, using noninvasive MRI, found that although CBF was unaltered, 50 g of glucose induced a time-dependent reduction in cerebral metabolic rate of O_2_ (Xu et al., 2015). A modulatory effect of sugar on CBFV therefore should not be ruled out. Additionally, the possibility that a combination of caffeine and sugar may exert a synergistic effect on CBFV should also be considered. Such an effect may be mediated by insulin, which has been shown to modulate CBF, although the direction of this effect is not yet clear (Page et al., 2013; Schilling et al., 2014).

Although weight correlated significantly with changes in CBFV, the effect was no longer present when men and women were investigated separately, and when a stepwise linear regression model was applied. Correlations between BMI and resting CBFV have been shown, but are controversial: a positive correlation was observed in women with anorexia nervosa (Kojima et al., 2005), while a negative correlation was found in patients with type-2 diabetes mellitus, hypertension, and stroke (Selim et al., 2008). Studies among subjects with anorexia nervosa allude to a possible influence of body weight on CBF, and show lower CBF in the bilateral anterior lobes (Kojima et al., 2005), as well as hypoperfusion in the anterior temporal lobe, which was not observed in a control group of non-anorexic women (Key et al., 2006). However, in the current study, although women appear to have a greater sensitivity to EDs, this effect was not explained by the influence of body weight (or etCO_2_) and therefore, is likely to be due to other factors yet to be determined.

A syncopal event can arise in response to any condition that diminishes CBF and oxygenation of regional cerebral tissue (Van Lieshout et al., 2003). Given that the incidence of neurocardiogenic syncope in women is twice that of men (Colman et al., 2004), this study has particular implications for women who appear to experience a greater reduction in CBFV than men in response to EDs. Future studies are warranted to investigate the role of ED consumption in patients suffering from vasovagal syncope and/or orthostatic intolerance.

Strengths of our study are the randomized and cross-over study design of the original studies, as well as the extensive data available on beat-to-beat derived cerebrovascular and cardiovascular parameters providing a high time-resolution. A limitation of the current study is the fact that data were analyzed retrospectively and that no blood-derived data on caffeine were available.

In conclusion, this retrospective analysis identified reduced CBFV in response to an ED, which differed significantly between genders, with women showing a greater reduction in CBFV than men. This gender effect was independent of body weight and etCO_2_, and persisted even after adjustment for differences in baseline CBFV. This study adds to the growing body of literature on the potential adverse effects of EDs, particularly the cerebrovascular effects, and reports a novel gender difference in CBFV in response to ED consumption. However, future intervention studies are warranted to determine the mechanisms of these gender differences.

## Author contributions

EG and JM designed research; CM and EG analyzed data and performed statistics; CM wrote the first draft of the manuscript; all authors were involved in the interpretation of data and contributed to the final draft; all authors read and approved the final version of the manuscript.

### Conflict of interest statement

The authors declare that the research was conducted in the absence of any commercial or financial relationships that could be construed as a potential conflict of interest.

## References

[B1] Ackerstaff R. G. Keunen R. W. van Pelt W. Montauban van Swijndregt A. D. Stijnen T. (1990). Influence of biological factors on changes in mean cerebral blood flow velocity in normal ageing: a transcranial Doppler study. Neurol. Res. 12, 187–191. 10.1080/01616412.1990.11739941 1979850

[B2] Addicott M. A. Yang L. L. Peiffer A. M. Burnett L. R. Burdette J. H. Chen M. Y.. (2009). The effect of daily caffeine use on cerebral blood flow: how much caffeine can we tolerate? Hum. Brain. Mapp. 30, 3102–3114. 10.1002/hbm.20732 19219847 PMC2748160

[B3] Ali F. Rehman H. Babayan Z. Stapleton D. Joshi D. D. (2015). Energy drinks and their adverse health effects: a systematic review of the current evidence. Postgrad Med. 127, 308–322. 10.1080/00325481.2015.1001712 25560302

[B4] Colman N. Nahm K. Ganzeboom K. S. Shen W. K. Reitsma J. Linzer M.. (2004). Epidemiology of reflex syncope. Clin. Auton. Res. 14, 9–17. 10.1007/s10286-004-1003-3 15480937

[B5] Grasser E. K. Dulloo A. G. Montani J. P. (2015). Cardiovascular and cerebrovascular effects in response to red bull consumption combined with mental stress. Am. J. Cardiol. 115, 183–189. 10.1016/j.amjcard.2014.10.017 25465941

[B6] Grasser E. K. Yepuri G. Dulloo A. G. Montani J. P. (2014). Cardio- and cerebrovascular responses to the energy drink Red Bull in young adults: a randomized cross-over study. Eur. J. Nutr. 53, 1561–1571. 10.1007/s00394-014-0661-8 24474552 PMC4175045

[B7] Higgins J. P. Yarlagadda S. Yang B. (2015). Cardiovascular complications of energy drinks. Beverages 1, 104–126. 10.3390/beverages1020104

[B8] Key A. O'Brien A. Gordon I. Christie D. Lask B. (2006). Assessment of neurobiology in adults with anorexia nervosa. Eur. Eat Disord. Rev. 14, 308–314. 10.1002/erv.696

[B9] Kojima S. Nagai N. Nakabeppu Y. Muranaga T. Deguchi D. Nakajo M.. (2005). Comparison of regional cerebral blood flow in patients with anorexia nervosa before and after weight gain. Psychiatry Res. 140, 251–258. 10.1016/j.pscychresns.2005.08.002 16288853

[B10] Kozik T. M. Shah S. Bhattacharyya M. Franklin T. T. Connolly T. F. Chien W.. (2016). Cardiovascular responses to energy drinks in a healthy population: the C-energy study. Am. J. Emerg. Med. 34, 1205–1209. 10.1016/j.ajem.2016.02.068 27162113

[B11] Lippi G. Cervellin G. Sanchis-Gomar F. (2016). Energy drinks and Myocardial ischemia: a review of case reports. Cardiovasc. Toxicol. 16, 207–212. 10.1007/s12012-015-9339-6 26320007

[B12] Marinoni M. Ginanneschi A. Inzitari D. Mugnai S. Amaducci L. (1998). Sex-related differences in human cerebral hemodynamics. Acta Neurol. Scand. 97, 324–327. 10.1111/j.1600-0404.1998.tb05961.x 9613563

[B13] Mathew R. J. Wilson W. H. (1985). Caffeine induced changes in cerebral circulation. Stroke 16, 814–817. 10.1161/01.STR.16.5.814 3901422

[B14] Mathew R. J. Wilson W. H. Tant S. R. (1986). Determinants of resting regional cerebral blood flow in normal subjects. Biol. Psychiatry 21, 907–914. 10.1016/0006-3223(86)90264-7 3091096

[B15] Nagai Y. Kemper M. K. Earley C. J. Metter E. J. (1998). Blood-flow velocities and their relationships in carotid and middle cerebral arteries. Ultrasound. Med. Biol. 24, 1131–1136. 10.1016/S0301-5629(98)00092-1 9833581

[B16] Page K. A. Chan O. Arora J. Belfort-Deaguiar R. Dzuira J. Roehmholdt B.. (2013). Effects of fructose vs glucose on regional cerebral blood flow in brain regions involved with appetite and reward pathways. JAMA 309, 63–70. 10.1001/jama.2012.116975 23280226 PMC4076145

[B17] Schilling T. M. Ferreira de sá D. S. Westerhausen R. Strelzyk F. Larra M. F. Hallschmid M.. (2014). Intranasal insulin increases regional cerebral blood flow in the insular cortex in men independently of cortisol manipulation. Hum. Brain Mapp. 35, 1944–1956. 10.1002/hbm.22304 23907764 PMC6869468

[B18] Selim M. Jones R. Novak P. Zhao P. Novak V. (2008). The effects of body mass index on cerebral blood flow velocity. Clin. Auton. Res. 18, 331–338. 10.1007/s10286-008-0490-z 18726054 PMC2600600

[B19] Svatikova A. Covassin N. Somers K. R. Somers K. V. Soucek F. Kara T. (2015). A randomized trial of cardiovascular responses to energy drink consumption in healthy adults. JAMA 314, 2079–2082. 10.1001/jama.2015.13744 26547226

[B20] Van Lieshout J. J. Wieling W. Karemaker J. M. Secher N. H. (2003). Syncope, cerebral perfusion, and oxygenation. J. Appl. Physiol. 94, 833–848. 10.1152/japplphysiol.00260.2002 12571122

[B21] Xu F. Liu P. Pascual J. M. Xiao G. Huang H. Lu H. (2015). Acute effect of glucose on cerebral blood flow, blood oxygenation, and oxidative metabolism. Hum. Brain Mapp. 36, 707–716. 10.1002/hbm.22658 25324201 PMC6869447

